# Evaluation of Mechanical Properties and Filler Interaction in the Field of SLA Polymeric Additive Manufacturing

**DOI:** 10.3390/ma16144955

**Published:** 2023-07-12

**Authors:** Petr Jirků, Jiří Urban, Miroslav Müller, Viktor Kolář, Vijay Chandan, Jaroslava Svobodová, Rajesh Kumar Mishra, Hafsa Jamshaid

**Affiliations:** 1 Department of Material Science and Manufacturing Technology, Faculty of Engineering, Czech University of Life Sciences Prague, Kamycka 129, 165 00 Prague, Czech Republic; jirkup@tf.czu.cz (P.J.); xurbj021@studenti.czu.cz (J.U.); muller@tf.czu.cz (M.M.); vkolar@tf.czu.cz (V.K.); vijay@tf.czu.cz (V.C.); 2 Faculty of Mechanical Engineering, University of Jan Evangelista Purkyně in Ústí nad Labem (UJEP), Pasteurova 3334/7, 400 01 Usti nad Labem, Czech Republic; jaroslava.svobodova@ujep.cz; 3 Faculty of Textile Engineering, National Textile University, Sheikhupura Road, Faisalabad 37610, Pakistan; hafsa@ntu.edu.pk

**Keywords:** 3D printing, polymer composite, carbon filler, cotton filler, cyclic loading, low cycle test

## Abstract

The paper deals with research focused on the use of fillers in the field of polymeric materials produced by additive technology SLA (stereolithography). The aim of the research is to evaluate 3D printing parameters, the mechanical properties (tensile strength, hardness), and the interaction of individual phases (polymer matrix and filler) in composite materials using SEM analysis. The tested fillers were cotton flakes and ground carbon fibres in different proportions. For the photosensitive resins, the use of cotton flakes as filler was found to have a positive effect on the mechanical properties not only under static but also under cyclic loading, which is a common cause of material failure in practice. The cyclic stress reference value was set at an amplitude of 5–50% of the maximum force required to break the pure resin in a static tensile test. A positive effect of fillers on the cyclic stress life of materials was demonstrated. The service life of pure resin was only 168 ± 29 cycles. The service life of materials with fillers increased to approximately 400 to 540 cycles for carbon fibre-based fillers and nearly 1000 cycles for cotton flake-based fillers, respectively. In this paper, new composite materials suitable for the use of SLA additive manufacturing techniques are presented. Research demonstrated the possibilities of adding cotton-based fillers in low-cost, commercially available resins. Furthermore, the importance of material research under cyclic loading was demonstrated.

## 1. Introduction

Stereolithography (SLA) is becoming an increasingly popular low-cost 3D printing technology among various users as it offers superior dimensional accuracy and overall better quality as compared to its closest and more widespread competitor, Fused Deposition Modelling (FDM)/Fused Filament Fabrication (FFF) [1,2].

Fused Deposition Modeling (FDM)/Fused Filament Fabrication (FFF) is currently a promising 3D printing technology that has received considerable research attention, particularly in the area of adding different fillers to filaments and recycling them [2,3].

There are concerns that the opaque nature of fillers added to photosensitive resins would reduce their photosensitivity. This could prevent perfect photocuring by preventing the fillers from absorbing UV radiation applied during the process [4]. Romero Ocana and Molina refuted this limiting factor of using filler in SLA technology. In addition, they demonstrated a significant effect of particle size on the final mechanical properties [4]. It is to be expected that as the amount of filler increases, there will also be problems during printing [5]. 

SLA (stereolithography) technology is a type of 3D printing that uses UV (ultraviolet) light to solidify liquid resin layer by layer, creating precise and highly detailed 3D printed objects [6,7,8]. The mechanical properties of UV resin, a material commonly used in SLA printing, play a critical role in determining the strength, stiffness, and overall performance of printed components [9]. A significant advantage over other 3D printing techniques is the absence of interfaces between layers. In addition to improving surface finishes, it has important implications for the mechanical properties of the individual layers. Further, it eliminates the micro-scale gaps and the associated overall reduction in crack-inducing inequalities [1]. 

Photosensitive resins (referred to as SLA resins) belong to the group of reactive resins (reactive plastics). The characteristic of a reactive resin is an irreversible chemical reaction while the material enters a cross-linked state [9]. The basic composition of resins is monomers, photoinitiators, and additives. The chemical mechanism used to cure photosensitive resins in SLA printers is called radical photopolymerization. When illuminated with UV light of the appropriate wavelength, light is absorbed by the photoinitiators. The photo-initiating agents initiate the curing reaction [9]. 

It should be emphasized that very few studies have used the SLA technique together with bio filler specifically to create new composite materials [10]. The mechanical properties of materials printed with SLA 3D printing are difficult to obtain from technical data sheets and, even when available, are often unreliable because they depend heavily on the specific printing parameters used [1]. Therefore, it is often necessary to perform the actual mechanical analysis.

While the inherent mechanical properties of UV resin can provide sufficient strength for many applications, further enhancement can be achieved with post-processing techniques or the introduction of reinforcing materials [11]. Subsequent curing, a process that involves exposing printed parts to additional UV light to further cure and harden the resin, can improve overall mechanical properties [12]. In addition, reinforcing the UV resin with additives or fillers such as carbon fibres, glass fibres, or metal particles can significantly increase its strength, stiffness, impact resistance, and other properties [12]. This process produces composite materials that exhibit increased mechanical strength and specific functional properties tailored to the requirements of the application [13].

Although the integration of fillers with SLA resins presents a number of opportunities, certain aspects must be kept in mind. First, the choice of filler and its concentration can significantly affect printability and other process parameters. Careful experimentation and optimization are required to achieve the desired properties without compromising print quality [12]. Nanofiller-containing resins offer the potential to improve properties such as thermal and mechanical, conductivity, magnetism, biocompatibility, and processing [14]. Many factors can complicate the introduction of composite resins into 3D printing systems. An important parameter for a printable resin is its viscosity. Small amounts of additives can have a large effect on the viscosity of the resin. If the viscosity is too high, the resin can be difficult to work with and may require longer curing times (the viscosity of unfilled resin is usually in the range of 150–200 mPa·s). Another common problem is dispersion. Many nanomaterials can prove incompatible with resins [12]. It can also be noted that components produced with SLA 3D printing are isotropic, but the addition of filler makes them partially anisotropic.

On the other hand, the advantage of adding filler to SLA resins is a potential improvement of the mechanical properties of the printed components [15].

In practice, polymeric materials are exposed to many complicated loading conditions, such as polymer matrix failure during deformation. Cracking is a phenomenon of the gradual accumulation of plastic deformation when materials and structures are exposed to cyclic loading with non-zero medium stress [16]. The accumulation of plastic deformation is an important aspect of fatigue damage [17,18]. The strength and service life of polymeric materials are reduced even at relatively low stress levels in cyclic fatigue [19,20,21]. This type of cyclic fatigue in a material is the most destructive form of mechanical failure, and it is the most common cause of degradation of these materials in practice [22]. The strength of composite materials is affected not only by the reinforcing component but also by the transfer of stress between the individual components of reinforcements and the matrix. That means the reinforcement should be properly impregnated by the matrix. The transfer of stress between the matrix and the reinforcement should be efficient since it has a significant effect on the service life of the material [19]. Cyclic tests are necessary for the practical use of such components [19,20,22].

This research is focused on the use of fillers in polymeric composite materials produced by additive SLA (stereolithography) technology. Composite materials based on reinforcement are among the promising materials for the future. There is considerable variability in the possibilities of using fillers in combination with polymeric materials and production technology. A great deal of research has been conducted in the field of FDM/FFF on printing parameters and filler options, but also on factors affecting material fatigue [23,24,25,26]. However, in the field of SLA 3D printing technology and the associated modification of resins with filler, there is still limited knowledge and a significant number of limiting factors related mainly to the functionality of printing with this modified material. 

This paper focuses on SLA 3D printing technology and the modification of a low-cost, widely available photosensitive resin, Coloured UV Resin, using a filler based on cotton flakes and ground carbon fibres in varying proportions. This work is focused on the development of a new photocurable polymer composite applicable in SLA 3D printing technology using a photosensitive resin as the polymer matrix. In this work, the mechanical properties (tensile strength, hardness) and the interaction of individual phases were investigated with SEM analysis. The resulting photocurable polymer composite should be useful for SLA 3D printing technology with promising results.

## 2. Materials and Methods

### 2.1. 3D Printer and Printing Parameters

**Figure 1:**
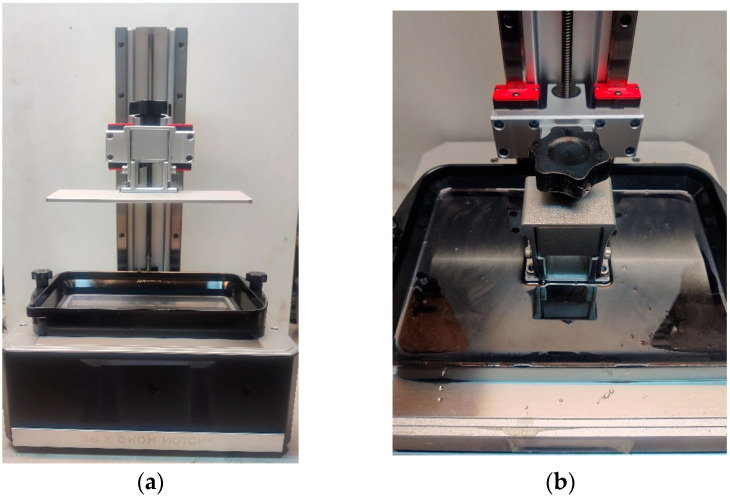
Anycubic Photon Mono X 6K (**a**); printing on SLA printer (**b**).

An MSLA Photon Mono X 6K (ANYCUBIC Technology Co., Ltd., Shenzhen, China) printer with a display resolution of 5760 × 3600 pixels was used to produce the samples (Figure 1). CHITUBOX software (version 1.9.4., CBD-Tech Co., Ltd., Shenzhen, China) was used for data preparation. The printing parameters are shown in Table 1. A longer exposure time of the initial layers is a common practice in SLA printing, increasing adhesion to the printing platform.

### 2.2. Resin, Fillers, and Their Preparation

Coloured UV resin (ANYCUBIC Technology Co., Ltd., Shenzhen, China) in transparent colour cured under 405 nm light was used for printing. The ground recycled carbon fibre used had a mean length of 200 ± 30 μm and a monofilament diameter of 7 ± 2 μm. The second filler used was cotton flakes with a specific gravity of 1.5 g·cm^−3^. The fibre length was 350 ± 150 μm and the fibre width was 17.5 ± 7.5 μm, as declared by the manufacturer. A summary of the materials produced is given in Table 2.

**Figure 2:**
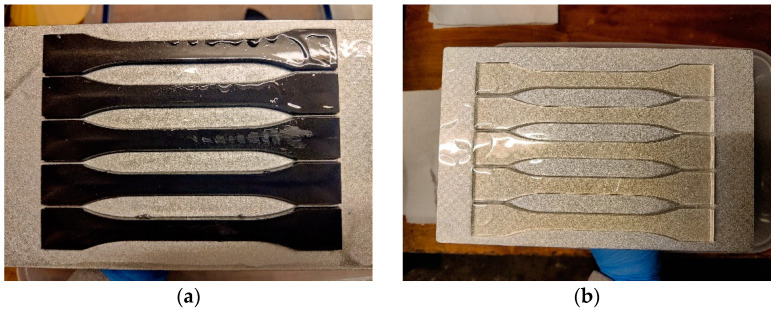
Printed test samples on the MSLA printer platform before cleaning and curing with carbon fibre (**a**) and with cotton flakes (**b**).

**Figure 3:**
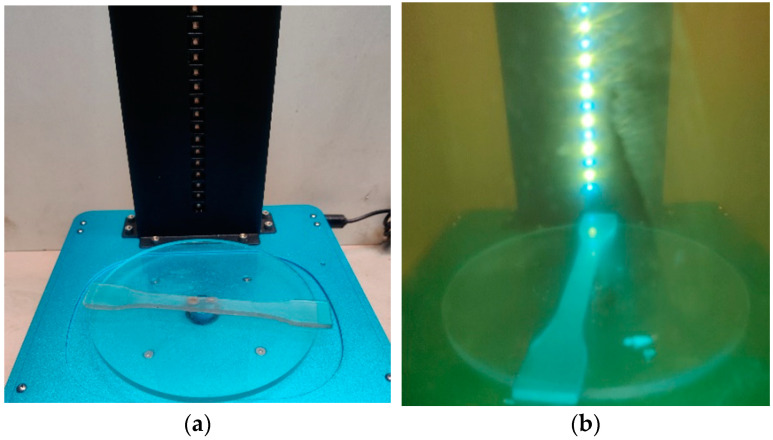
Sample ready for curing on Anycubic Wash & Care facility platform (**a**), sample during the curing process (**b**).

After printing (Figure 2), the samples were cleaned of excess resin in isopropanol (INCHEMA s.r.o., Prague, Czech Republic). The samples were then cured by exposure to UV light for 2 min (Figure 3) in the Anycubic Wash & Care facility (ANYCUBIC Technology Co., Ltd., Shenzhen, China).

**Figure 4:**
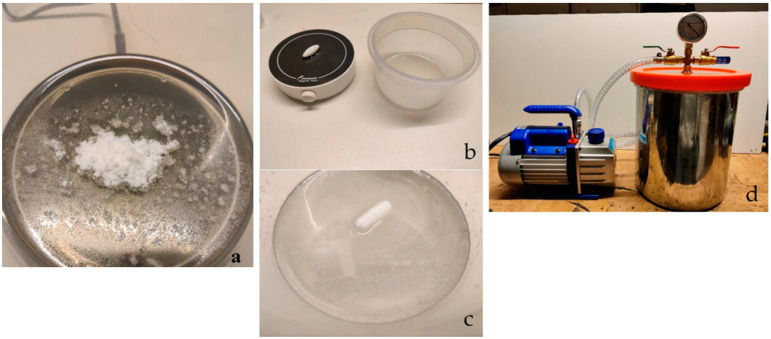
Process of mixing resin with filler: (**a**) filler in resin before mixing, (**b**) magnetic stirrer, (**c**) mixed mixture, (**d**) vacuum chamber.

For the preparation of resin with filler, a certain amount of resin was weighed, and filler was added to it in a specified ratio (Figure 4a). A magnetic stirrer was used to achieve the greatest possible homogeneity of the suspension (Figure 4b). The mixing was performed using a rotating magnetic stirrer inside the suspension (Figure 4c). The stirring was carried out for 10 min. The air bubbles formed after mixing were removed using a vacuum chamber (Figure 4d).

The mixed matrix behaves stably during the printing of the test samples. There was no clumping/agglomeration of the filler in the resin. This is due to the repeated movement of the printing platform in the *z*-axis during printing according to the parameter set in Table 1 (“Lifting Distance”).

### 2.3. Test Bodies/Samples

**Figure 5:**
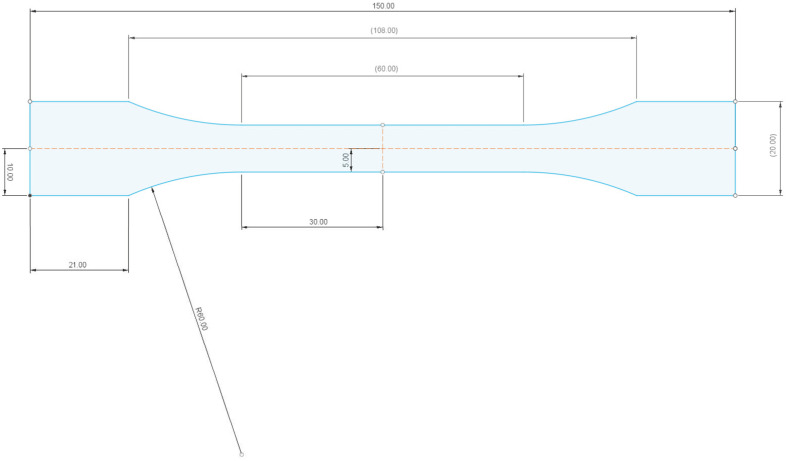
Sketch of test body/sample in Fusion 360 software (version 2.0.15.050).

The design of the test bodies/samples was implemented using Fusion 360 software (version 2.0.15.050, Autodesk, Inc., San Francisco, CA, USA). The test body was designed according to the standard EN ISO 527-2 (see Figure 5). A sketch of the test specimen with the required dimensions was drawn and the required thickness was defined using the “eject” function.

### 2.4. Testing of Mechanical Properties

**Figure 6:**
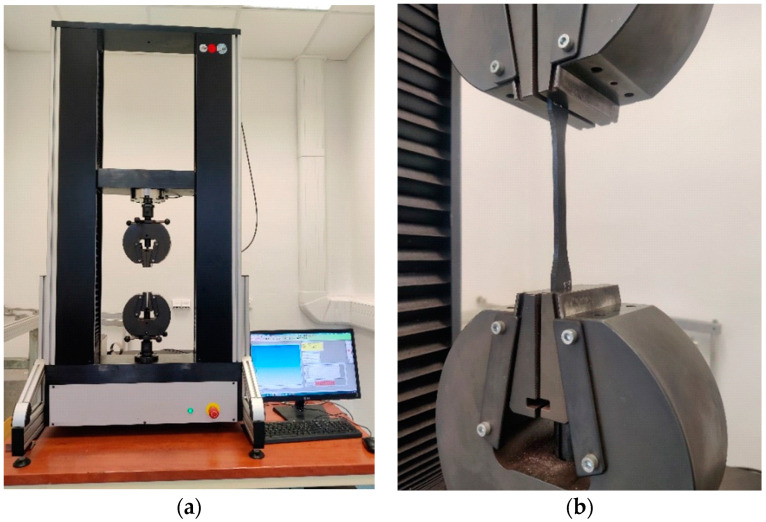
LabTest 5.50ST test rig (**a**) and detail of test body between jaws (**b**).

The testing of mechanical properties was carried out on the universal testing machine LABTest 5.50 ST (LABORTECH s.r.o., Opava, Czech Republic), with the measuring unit AST KAF 50 kN (LABORTECH s.r.o., Opava, Czech Republic) and the evaluation software Test & Motion (version 4.5.0.15, LABORTECH s.r.o., Opava, Czech Republic). Figure 6 presents the test rig LabTest 5.50ST (a) and a detail of the test body between the jaws (b). 

The static test speed was set to 10 mm·min^−1^. The cycling test was set to 1000 cycles according to the requirement of the research sponsor and at the same time due to the comparison with already published results on 3D printing using FDM/FFF technology with PLA-based filament along with synthetic and biological fillers.

**Figure 7:**
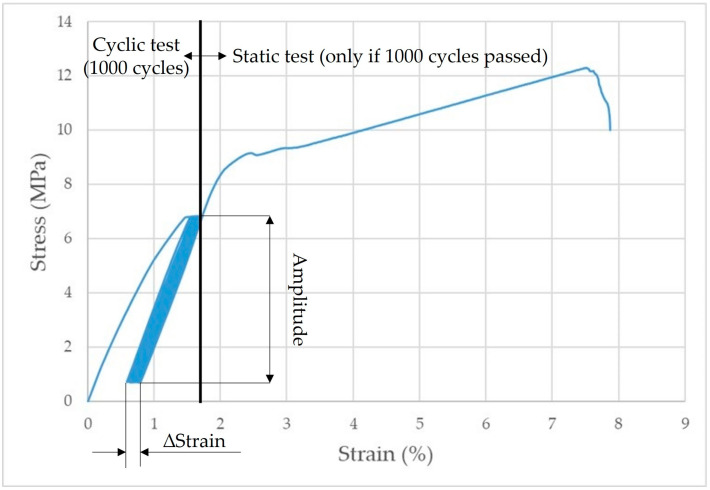
Principle of cyclic test of samples.

Therefore, a low-cycle stress test was selected. The methodology for testing the mechanical properties under cyclic loading involves determining a reference value obtained during the static tensile test from the RC test bodies. The reference value corresponds to the maximum force required for complete failure of the test body during the static tensile test. Cyclic testing constitutes setting the amplitude obtained as a percentage of the reference value (% of Fmax). The amplitude was set as 5–50% of the reference value. The test bodies were stressed for 1000 cycles at a rate of 10 mm·min^−1^. After the completion of 1000 cycles, a static tensile test was automatically followed until complete failure of the bonded joint at a rate of 0.6 mm·min^−1^. The static test was only performed when the 1000th cycle was completed. Otherwise, the test was terminated. The time delay at the lower and upper limits was set to 0.1 s. The ΔStrain value expresses the visco-elastic behaviour of the material. Figure 7 shows the principle of low cycle loading of the test samples.

**Figure 8:**
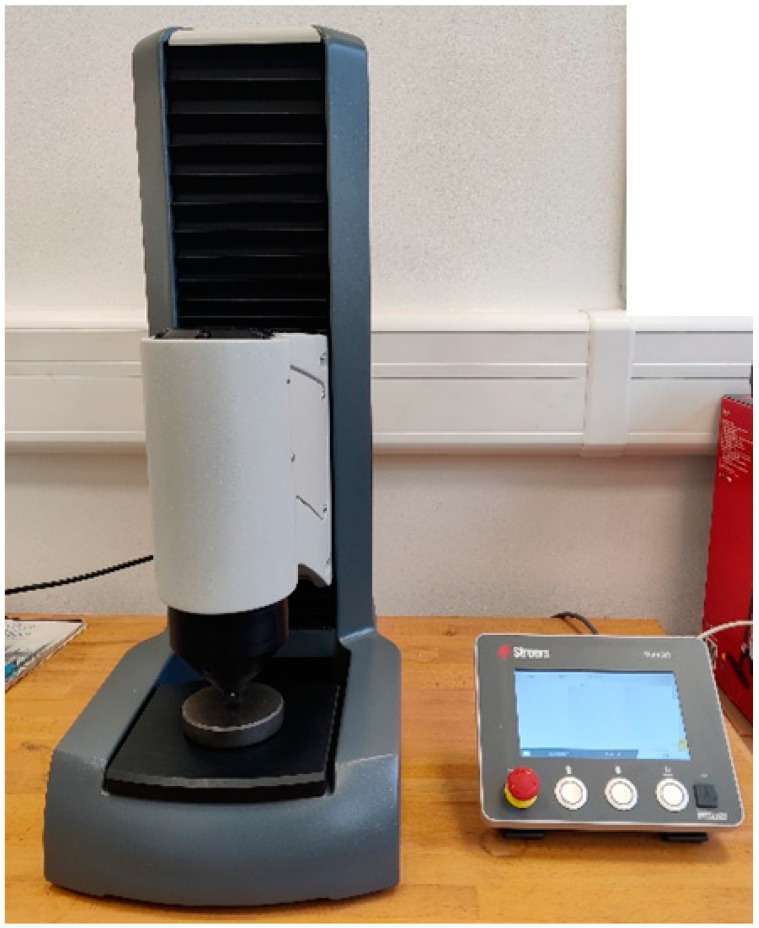
Hardness tester DuraJet G5.

In addition to the tensile test, a hardness test was performed using a DuraJet G5 hardness tester (Struers GmbH, Roztoky u Prahy, Czech Republic), see Figure 8, based on ČSN EN ISO 2039-1 [26]. A hardened steel ball with a diameter of 5 mm, an initial load of 9.8 N, and a test load of 132 N was used for the hardness test. This is because the first 6 layers on the printing platform are subjected to a longer exposure time; see printing parameters in Table 1. 

### 2.5. Statistical Evaluation of the Measured Data

Evaluation of the experimental data was performed with analysis of variance, i.e., ANOVA F-test in STATISTICA (version 14.0.0.15, StatSoft CR, Prague, Czech Republic). The statistical dependence at the 0.05 significance level among the RC standard and the other experimental variants was evaluated. The null hypothesis H_0_ presenting a statistically insignificant difference in mechanical properties among the RC and the other experimental variants was established (*p* > 0.05). Hypothesis H_1_ rejects the null hypothesis H_0_ and presents a statistically significant difference in mechanical properties among the RC and the other experimental variants (*p* < 0.05).

### 2.6. Scanning Electron Microscopy (SEM) Analysis

SEM analysis was performed using a TESCAN VEGA 3 XMU (TESCAN ORSAY HOLDING a.s., Brno, Czech Republic). The microscopic samples were coated with gold using Quorum Q150R ES Plus (Quorum Technologies–Judges House, Laughton, UK).

## 3. Results and Discussion

### 3.1. Mechanical Test—Static and Cyclic Tensile Test

This research is concerned with the mechanical properties of polymer-based composite materials, which were fabricated using SLA 3D printing technology. Affordable SLA 3D printing materials do not achieve good mechanical properties when compared to FDM/FFF printing. Using filler materials is one way to overcome this limitation.

**Figure 9:**
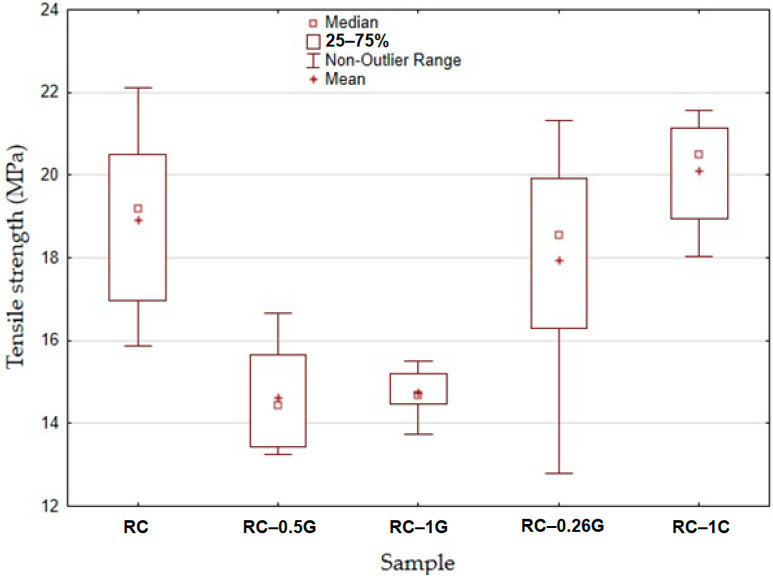
Static tensile test results—strength.

Figure 9 presents the tensile strength of the samples. For the static tensile test, a total of 8 measurements were carried out for each material variant. The RC material exhibited a tensile strength of 18.91 ± 2.05 MPa in the static tensile test. The addition of carbon fibres (RC-0.5G) resulted in an average reduction of 22.37% in interfacial tensile stress compared to RC. RC-1G exhibited a tensile strength of 14.74 ± 1.04 MPa, which is 22.1% less than the value for RC. At the same time, RC-0.5G and RC-1G showed a significant reduction in standard deviation, indicating greater homogeneity of the specimens.

In the case of printing with ground carbon fibre, there were problems with SLA 3D printing. These problems were mainly due to technical restrictions during printing, including the lack of UV light curing, which was caused by the local deposition of a large number of carbon fillers that could not be cured with the printing setup used. This deposition was caused by the lack of homogeneity of the suspension.

In contrast, RC-0.25C did not significantly affect the ultimate tensile strength. In the case of RC-1C, there was a 6.3% increase in tensile strength compared to RC (20.1 ± 1.2 MPa). The obtained results confirmed the conclusions of other researchers who found that certain fillers can lead to an increase in the strength of UV resins [12]. This trend was evident for cotton-based fillers. 

**Figure 10:**
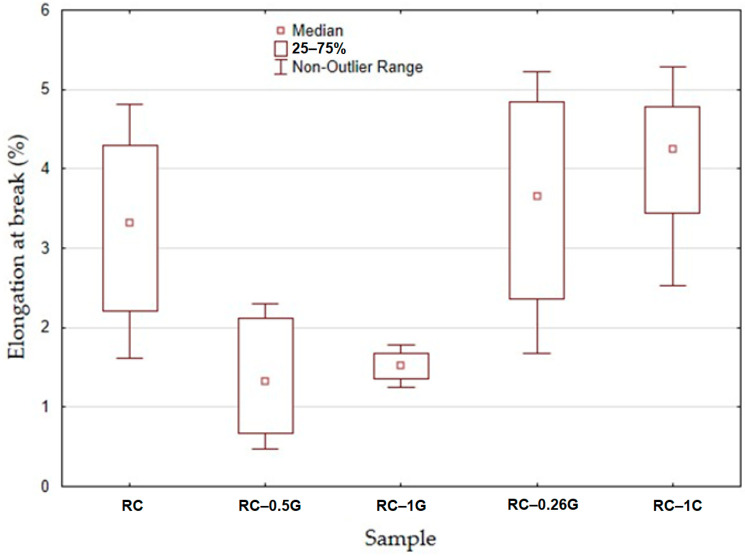
Static tensile test results—elongation at break.

Figure 10 presents the elongation at break for the samples. It can be seen that the RC material exhibited an elongation at break value of 3.26 ± 1.1%. The RC-0.5G and RC-1G materials showed a decrease in elongation at break (1.38 ± 0.73% and 1.27 ± 0.58%), respectively. For RC-0.25C and RC-1C materials, there was a slight increase in elongation at break (3.61 ± 1.22% and 4.1 ± 0.86%), respectively.

The results of the statistical evaluation of the mechanical properties after static tensile testing of individual materials are shown in Table 3.

Table 3 shows that RC-0.5G and RC-1G materials showed a statistically significant effect, i.e., the filler significantly reduced the strength of the materials. The RC-0.25C and RC-1C materials showed a slight increase in strength, but this increase is not statistically significant. 

Mechanical tests were complemented by cyclic loading tests according to user-defined specifications with a view to practical application. The conclusions from the cyclic loading tests are very significant, given that a large part of product failures was caused by cyclic loading of varying intensity and number of cycles. Generally, research on materials tends to focus only on static tests; however, for practical applications, materials also have to be tested under cyclic stresses, which are largely responsible for their failure. The results of the cyclic tests are shown in Table 4.

Table 4 shows that the RC material did not withstand the specified number of 1000 cycles. The average value of completed cycles was 168 ± 29. It is, therefore, clear that the RC material exhibits a reduced resistance to cyclic stresses of 5–50% amplitude. The RC-0.5G material showed an average number of completed cycles, 404 ± 139. Thus, there is a positive effect of the added filler on the cyclic stress life. RC-1G did not last the specified number of 1000 cycles. However, it is noticeable that there was a slight increase in the number of completed cycles compared to RC-0.5G. The RC-0.25C material showed a significantly higher number of completed cycles for two tests, 912 and 936. One test completed the specified number of 1000 cycles. Thus, a positive effect of the cotton flake filler on the cyclic life of the material is evident. For RC-1C, two complete tests, i.e., 1000 cycles, were completed. One test failed prematurely at 963 cycles. The above results indicate that the cotton flake filler significantly (positively) affected the cyclic loading life of the materials. This is due to the fact that the stress applied to the material is uniformly distributed to the filler within the composite system [27]. Based on the above results, it can be confirmed that cyclic loading can negatively affect the service life of polymeric material [28,29], especially for RC material. There is an assumption of microcrack propagation due to cyclic loading and the magnitude of its amplitude [30]. In polymeric materials, the response to cyclic loading is primarily visco-elastic, but microcracking can also occur at higher values of cyclic loading [31]. If cyclic loading exceeds the elastic limit, there is an accumulation of plastic deformation, which is then the cause of the destruction of the test specimen [29]. The results show that material failure can occur even with a relatively low number of completed cycles [32]. Zhang et al. [18] reached a similar conclusion in their research on adhesive bonds.

**Figure 11:**
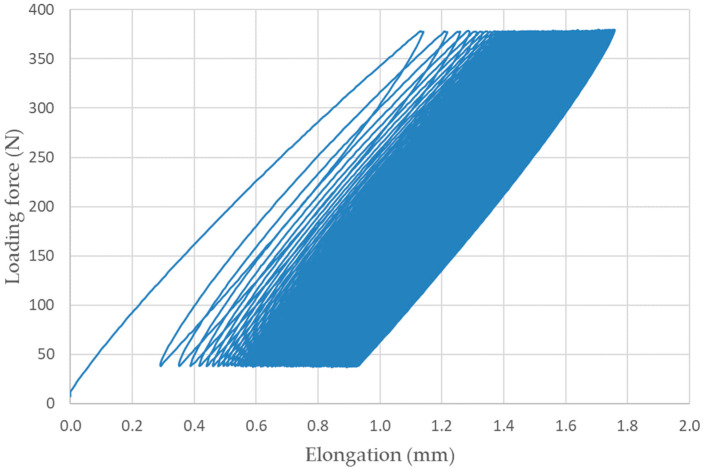
Quasi-static curve of RC material at cyclic loading 5–50%, 155 cycles completed.

Figure 11 shows an example of the quasi-static curve of the RC material that withstood 155 cyclic stress cycles with an amplitude of 5–50%.

**Figure 12:**
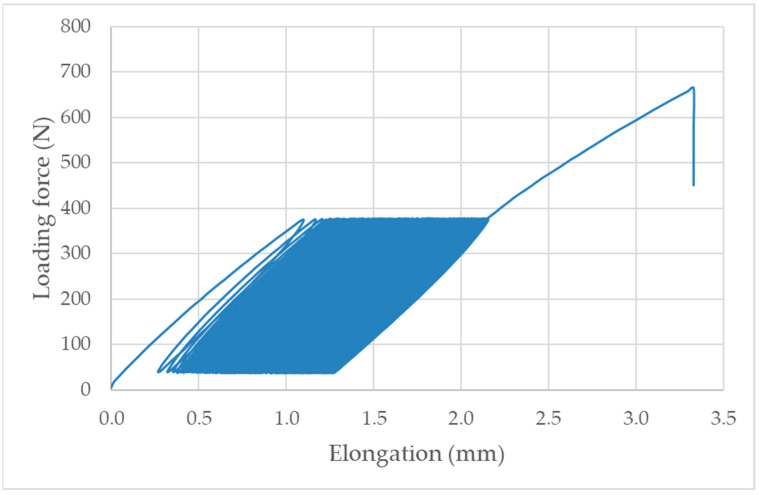
Quasi-static curve of RC-0.25C at 5–50% cyclic loading, 1000 cycles completed + subsequent static test.

Figure 12 shows the complete quasi-static curve of RC-0.25C and the static test that followed the successful completion of 1000 cycles.

Based on quasi-static curves (hysteresis loops), the visco-elastic behaviour of materials can be analyzed on the basis of accumulated stress and associated deformation. These factors cause the displacement of the hysteresis loops. Senatov et al. [33] stated that the displacement of the hysteresis loop makes it possible to evaluate the value of the accumulated deformation for the respective cycle or the number of cycles. Change in the width and area of the hysteresis loop allows us to evaluate the value of the reversible deformation that was dissipated during the cyclic loading [33]. Unfortunately, in this case, the evaluation based on hysteresis loops was not possible due to premature failure of the materials under cyclic loading (before the completion of 1000 cycles), which is presented in Table 4.

### 3.2. Hardness Test

The results of the hardness test are summarised in Table 5. The values in the table are given in the format mean ± standard deviation. A total of 6 measurements were performed for each material. For RC-1C variants, the hardness increased from the original 48.3 to 58.4, an increase in hardness of 20.93%. The hardness of a material is a very important factor affecting the usability of printed products. The increase in hardness value is a positive trend, although the hardness of these photosensitive resins is low compared to materials commonly used in FDM/FFF 3D printing. Examples include HB5/132 for PLA 240 to 290 and ABS 67 to 83. By modifying the RC filler, the hardness approached the value of the commonly used ABS plastic.

The experimental results refuted the concerns related to the opacity of the added fillers and the reduction in their photosensitivity, which would have prevented the perfect photocuring mentioned in the literature [4].

There are concerns that the opaque nature of fillers added to photosensitive resins would reduce their photosensitivity. This could prevent perfect photocuring by the fillers not pre-absorbing the UV radiation applied during the process. Romero Ocana and Molina refuted this limiting factor for the use of fillers in SLA technology. In addition, they demonstrated a large effect of particle size on the resulting mechanical properties [4]. It can be expected that printing problems will occur as the amount of filler increases [5].

### 3.3. SEM Analysis of Carbon Fibre and Cotton Fibre/Flakes 

**Figure 13:**
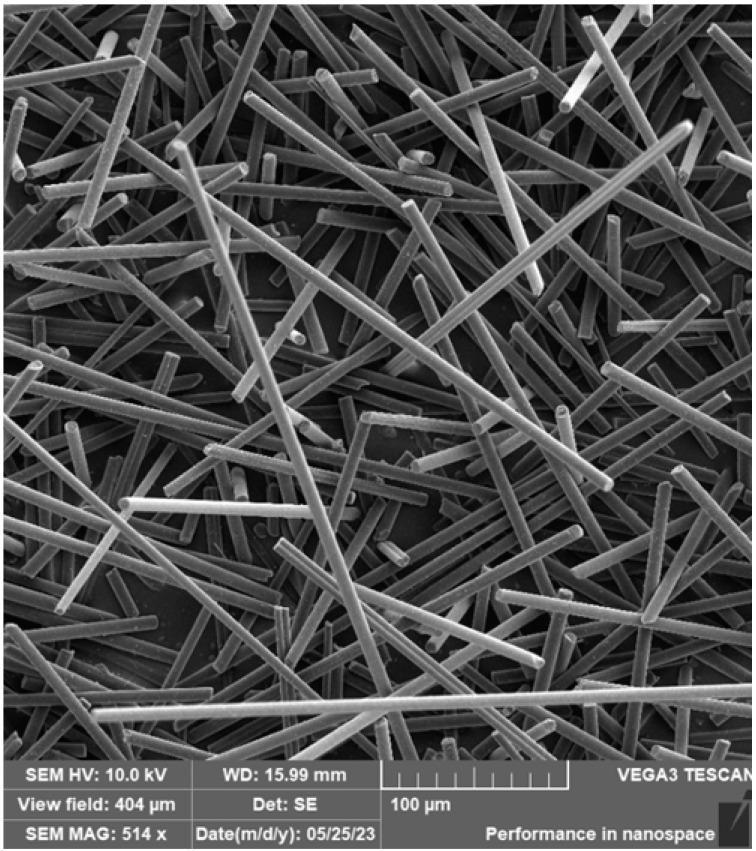
SEM of carbon fibre.

**Figure 14:**
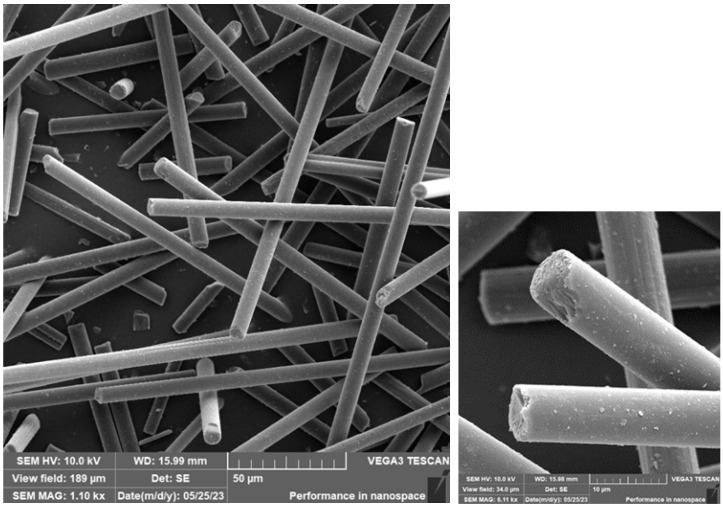
SEM of carbon fibre—detail.

The carbon fibres were deposited on an aluminium sample holder equipped with a carbon target. Subsequently, the carbon fibres were sputtered with a 10 nm layer of Au. Figure 13 and Figure 14 document the carbon fibre structure at different magnifications. From the SEM analysis, it is apparent that the fibres have different lengths, a regular cylindrical shape, and a smooth surface. The fibres were measured in their diameter and length. The length of the fibres (that could be measured as a whole) was, on average, approx. 155 μm, the diameter of the fibres was approx 7 μm.

**Figure 15:**
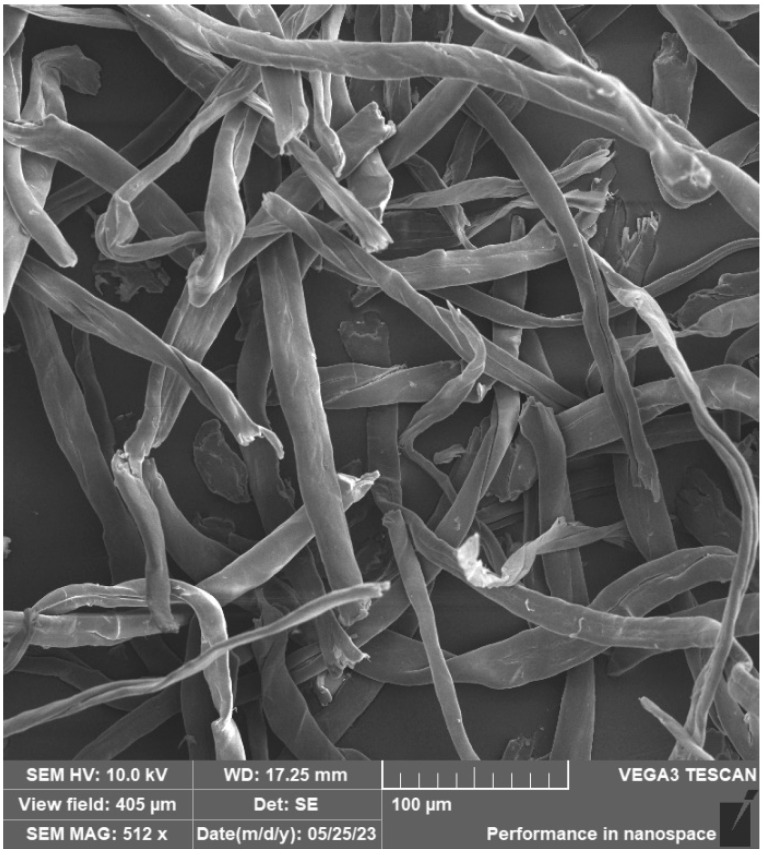
SEM of cotton fibre/flakes.

**Figure 16:**
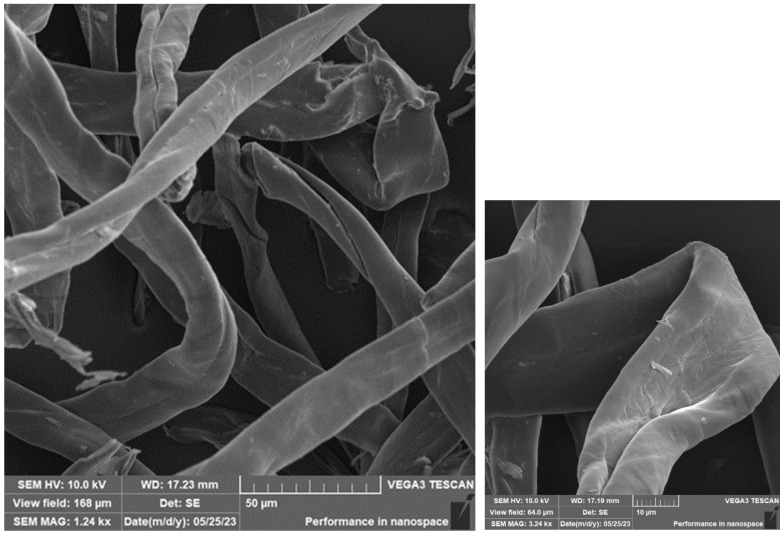
SEM of cotton fibre/flakes—detail.

Cotton fibres/flakes were placed on an aluminium sample holder with a carbon target in the same way as carbon fibres and sputtered with a 10 nm thick layer of Au. Cotton fibres are documented in Figure 15 and Figure 16. It was not possible to measure the length of this type of fibre. Only the thickness of the fibres was measured, which had an average diameter of 20 μm. The fibres have an irregular shape and length, and the surface of the fibres is smooth.

**Figure 17:**
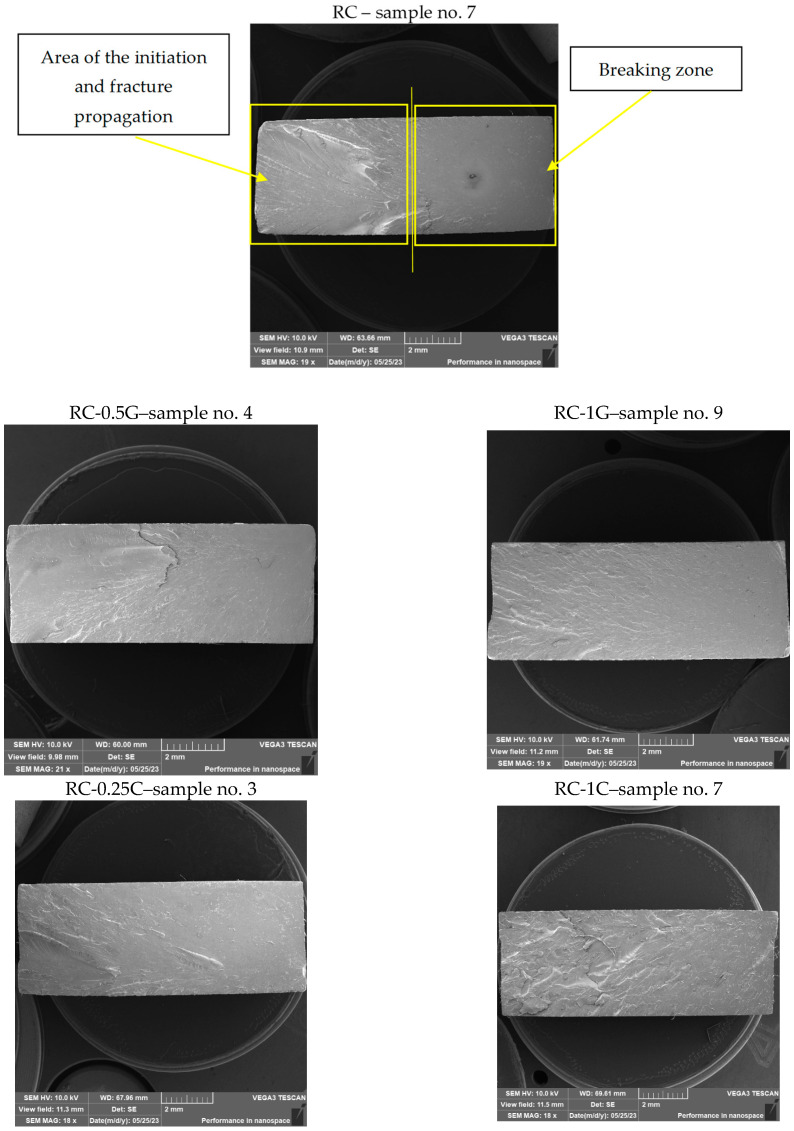
Fractographic SEM analysis of fracture surfaces.

Fractographic SEM analysis of the fracture surface of experimental samples is shown in Figure 17.

The samples were prepared from printed specimens after static tensile testing before SEM analysis of the fracture surface. An approximately 1.5 cm long part of the test sample with a fracture was cut off from the test body with a precision metallographic saw so as not to damage or contaminate the fracture surface. The sample thus prepared for observation was then placed on an Al holder of experimental samples for SEM, fixed with a carbon target, and the fracture surface was sputtered with a 10 nm layer of Au.

Essentially, two areas can be defined, namely the origin and development of the fracture and its breaking zone on the fracture surfaces of all samples (Figure 17). The area of development is characterized by the fragmentation of the fracture surface, while the fracture surface is smooth without significant relief of the fracture. This breaking starts approximately in the middle of the cross-sectional area of the sample.

**Figure 18:**
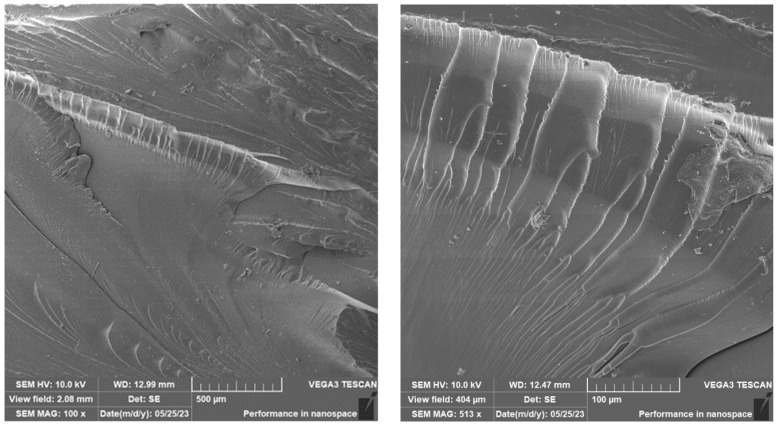
Fractographic SEM analysis of fracture surface—sample RC.

**Figure 19:**
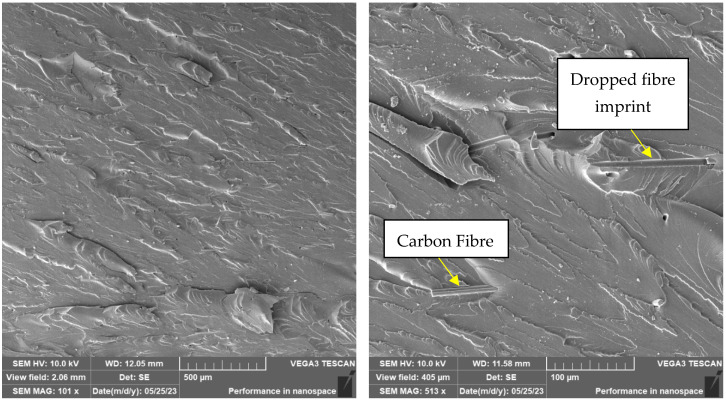
Fractographic SEM analysis of fracture surface—sample RC-1G.

**Figure 20:**
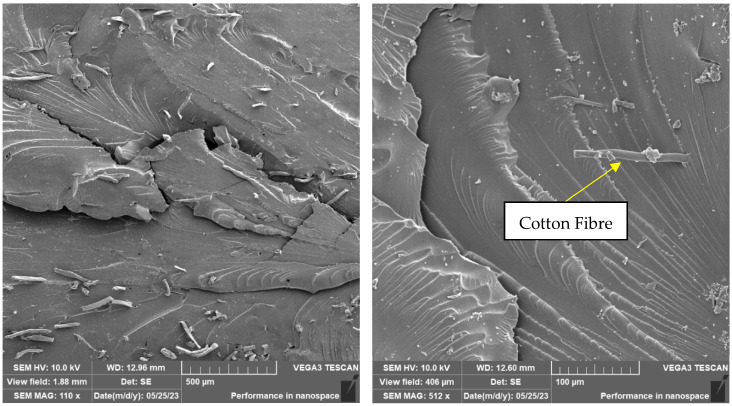
Fractographic SEM analysis of fracture surface—sample RC-1C.

A brittle fracture with line morphology was observed in all samples, as documented in Figure 18, Figure 19 and Figure 20.

In the case of the photosensitive resin with ground carbon fibres, disordered filler can be observed (Figure 18 and Figure 20). This disorder is manifested by the different spatial orientations of the individual fibres. It is also evident from Figure 19 that the fracture surface has been stripped of all fibres and cavities have been left behind. This is most likely due to imperfect wetting of the carbon fibres and poor interfacial bonding during the curing process. 

The presence of individual types of fibres can be observed on the fracture surface of sample RC-1G, Figure 19. There are fibres embedded in the matrix, but also fibres lying freely on the fracture surface. Carbon fibre imprints and holes from where the fibres were pulled can also be observed on the fracture surface. It is the same with cotton fibre (sample RC-1C, Figure 20). The fillings are pulled out of the matrix and can be observed on the fracture surface. However, fibre imprints were not observed in this case. The fibres are more flexible and better adhered to the matrix. Therefore, they do not fall out of the matrix and do not leave imprints like carbon fibres. However, there are holes on the fracture surface after the fibres have been pulled out.

**Figure 21:**
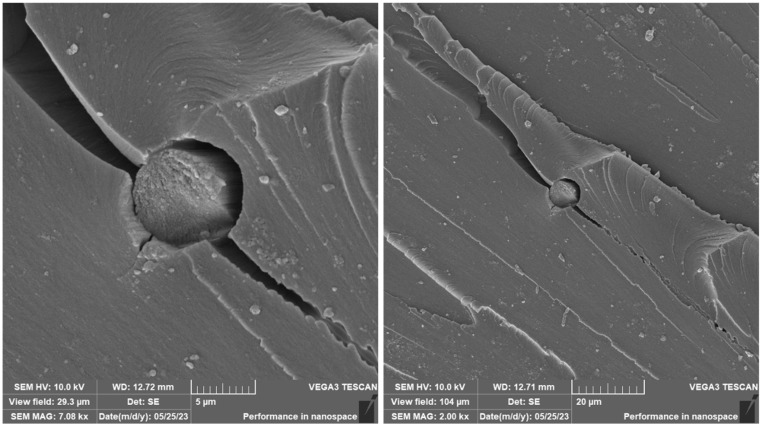
Fractographic SEM analysis of fracture surface—sample RC-1C (sample no. 9).

**Figure 22:**
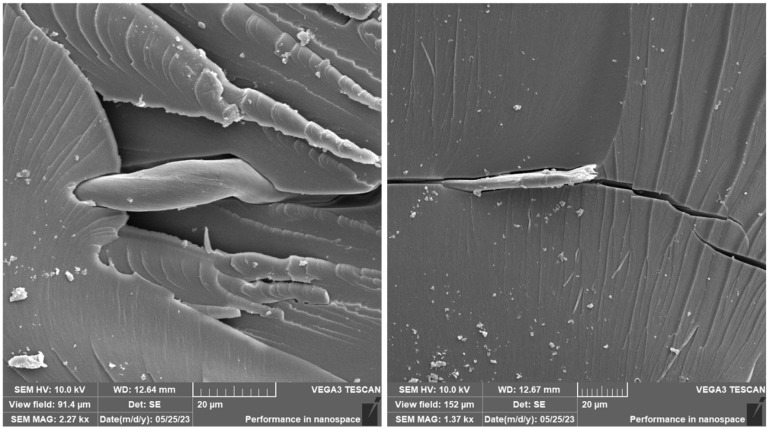
Fractographic SEM analysis of fracture surface—sample RC-1C (sample no. 8).

The difference between the samples filled with carbon and cotton fibres can be observed in Figure 21 and Figure 22, which document the detail of the filling on the fracture surface.

As can be seen from the documentation of the fracture surface, the filling in the form of carbon or cotton fibres contributes to the formation and development of the cracks. These fillings, therefore, contribute to the reduction in the mechanical properties of the composite material.

After SEM analysis of the tested samples, it can be concluded that no pores were detected on the surface. Pore formation during the 3D printing process is a substantial problem that has been reported in the literature [4,34,35].

The photosensitive resins did not have suspicious aggregates of fillers in the fracture surfaces. This implies that a certain degree of homogeneity was achieved. At the same time, it was not possible to observe a significant amount of air bubbles, which could have a negative effect on the mechanical properties. It can, therefore, be concluded that the process of removing air bubbles by means of the vacuum chamber was efficient during the preparation of the materials. The cotton fibre residue showing a simple shape and structure can be observed in Figure 22. These fibres are shown in more detail in Figure 15 and Figure 17. According to SEM analysis, it can be concluded that during the mixing of the filler and resin, there was a uniform dispersion. There are no suspicious aggregates of filler in the fracture surfaces of the test samples. In the SEM analysis, for example, different spatial orientations of the individual carbon fibres can be observed.

With the development of additive technologies, it is necessary to carry out intensive research in the field of modification of printing materials, i.e., to take into account the influence and the possibility of the effective use of natural fillers, printing parameters, the possibility of recycling, and testing of the resulting mechanical properties taking into account the specific details of the production technology and the application area.

Currently, the price of printing materials for SLA technology is much higher when compared to materials for FDM printers. At the same time, commercial photopolymers do not achieve satisfactory mechanical properties. This research focused on the use of fillers in commercial printing materials for SLA 3D printers in order to modify mechanical properties and print quality and, secondarily, to reduce the input cost of consumables.

## 4. Conclusions

In this research, new composite materials suitable for use in stereo-lithography SLA additive manufacturing techniques are presented. Research demonstrated the possibilities of adding cotton-based fillers to low-cost, commercially available resins. For photosensitive resins, the filler was shown to have a positive effect on mechanical properties. For this reason, further investigation of the mechanical properties of fillers based on secondary raw materials can be recommended, consistent with an environmental approach. The results of the experiment refuted the concerns related to the prevention/reduction of photocuring due to the opacity of the added fillers. This aspect influencing further research activities related to the modification of photosensitive resins for 3D printing with SLA technology was refuted, especially in the use of natural fillers. Affordable SLA 3D printing materials do not achieve good mechanical properties when compared to FDM/FFF printing. Using filler materials is one way to overcome this limitation. Specifically, the cotton flake filler was shown to have a positive effect on the mechanical properties of photosensitive resins.

The following significant conclusions were drawn from the experimental results.

- The addition of a carbon-based filler resulted in a reduction in tensile strength.
- The addition of cotton flake filler resulted in a slight improvement in tensile strength.
- The addition of carbon-based and cotton flake-based filler increased the number of completed cycles during low-cycle tests of tested materials.

## Figures and Tables

**Table 1:** 3D printing parameters and settings.

Parameter Name	Parameter Value
Layer height	0.05 mm
Exposure Time	2 s
Bottom Exposure Time	23 s
Bottom Layer Count	6
Anti-aliasing	1
Lifting Distance	8 mm
Lifting Speed	2 mm/s

**Table 2:** Variants of materials and their marking.

Matrix	Filler	Proportion of Filler with Matrix [wt.%]	Marking
UV Resin Clear	-	-	RC
UV Resin Clear	Carbon fibre	0.5	RC-0.5G
UV Resin Clear	Carbon fibre	1	RC-1G
UV Resin Clear	Cotton Flakes	0.25	RC-0.25C
UV Resin Clear	Cotton Flakes	1	RC-1C

**Table 3:** Results of Statistical Evaluation of Static Tensile Testing of Individual Materials.

	P-Parameter				
Material	RC	RC-0.5G	RC-1G	RC-0.25C	RC-1C
Tensile strength (MPa)	-	0.0003	0.0002	0.4570	0.2058
Elongation at break (%)	-	0.0021	0.0010	0.9115	0.1353

**Table 4:** Cyclic test results.

5–50%	Number of Completed Tests (1000 Cycles)/Total Number of Tests	Number of Completed Cycles
RC	0/3	168 ± 29
RC-0.5G	0/3	404 ± 139
RC-1G	0/3	541 ± 54
RC-0.25C	1/3	2 tests—912 and 936 cycles 1 test—1000 cycles
RC-1C	2/3	1 test—963 cycles 2 tests—1000 cycles

**Table 5:** Measured hardness values HB 5/132 for SLA printing materials.

Material	HB 5/132
RC	48.29 ± 1.96
RC-0.5G	40.36 ± 2.45
RC-1G	49.78 ± 2.35
RC-0.25C	59.58 ± 3.22
RC-1C	58.4 ± 4.21

## Data Availability

Not applicable.
